# US children of minority race are less likely to be admitted to the pediatric intensive care unit after traumatic injury, a retrospective analysis of a single pediatric trauma center

**DOI:** 10.1186/s40621-021-00309-x

**Published:** 2021-04-12

**Authors:** Katherine N. Slain, Morgan A. Wurtz, Jerri A. Rose

**Affiliations:** 1 Department of Pediatrics, Division of Pediatric Critical Care, University Hospitals Rainbow Babies & Children’s Hospital, 11100 Euclid Avenue, Mailstop RBC 6010, Cleveland, OH 44106 USA; 2 Case Western Reserve University School of Medicine, Cleveland, OH USA; 3 Department of Pediatrics, Division of Pediatric Emergency Medicine, Nationwide Children’s Hospital, 700 Children’s Drive, Columbus, OH 43205 USA; 4 Department of Pediatrics, Division of Pediatric Emergency Medicine, UH Rainbow Babies & Children’s Hospital, 11100 Euclid Avenue, Mailstop RBC 6002, Cleveland, OH 44106 USA

**Keywords:** Pediatric trauma, Critical care, Racial disparities, Hospital utilization

## Abstract

**Background:**

The public health impact of pediatric trauma makes identifying opportunities to equalize health related disparities imperative. The influence of a child’s race on the likelihood of admission to the pediatric intensive care unit (PICU) is not well described. We hypothesized that traumatically injured children of minority race would have higher rates of PICU admission, compared to White children.

**Methods:**

This was a retrospective review of a single institution’s trauma registry including children ≤18 years of age presenting to the emergency department (ED) whose injury necessitated pediatric trauma team activation at a Level 1 Pediatric Trauma Center from July 1, 2011 through June 30, 2016. Demographics, injury characteristics and hospital utilization data were collected. Race was categorized as White or racial minority, which included patients identifying as Black, Hispanic ethnicity, Native American or “other.” The primary outcome measure was admission to the PICU. Chi square or Mann Whitney rank sum tests were used, as appropriate, to compare differences in demographics and injury characteristics between those children who were and were not admitted to the PICU setting. Variables associated with PICU admission in univariate analyses were included in a multivariate analysis. Data are presented as median values and interquartile ranges, or numbers and percentages.

**Results:**

The median age of the 654 included subjects was 8 [IQR 4–13] years; 55.2% were a racial minority. Nine (1.4%) children died in the ED and 576 (88.1%) were admitted to the hospital. Of the children requiring hospitalization, 195 (33.9%) were admitted to the PICU. Children admitted to the PICU were less likely to be from a racial minority group (26.1% vs 42.5%, *p* < 0.001). After adjusting for age and injury characteristics in a multivariable analysis, racial minority children had a lower odds of PICU admission compared to White children (OR 0.492 [95% C.I. 0.298–0.813, *p* = 0.006]).

**Conclusions:**

In this retrospective analysis of traumatically injured children, minority race was associated with lower odds of PICU admission, suggesting that health care disparities based on race persist in pediatric trauma-related care.

**Supplementary Information:**

The online version contains supplementary material available at 10.1186/s40621-021-00309-x.

## Introduction

Injury is a leading cause of morbidity and mortality among children in the United States (US). (CDC Childhood Injury Report, 2008; Tracy et al., 2013) Approximately 4% of injured children require hospital admission, at an estimated annual total cost of $674 million in 2016. (Avraham et al., 2019; Moore et al., 2019) Of these hospitalized children, the most critically injured will require admission to the pediatric intensive care unit (PICU). Critical traumatic injuries requiring PICU care are associated with high morbidity, with up to 17% of children discharged with a new moderate to severe disability. (Flynn-O'Brien et al., 2017; Ahmed et al., 2019) The public health impact of critical injuries in children make optimizing prevention, treatment, and recovery imperative, including identifying opportunities to equalize health related disparities.

Multiple factors including systemic racism and discrimination, socioeconomic status, neighborhood condition, and health care access—collectively termed social determinants of health—are known to significantly impact childhood wellbeing. (Garner et al., 2012) It is well established that social determinants affect outcomes, including mortality, related to pediatric trauma. However, less is known about the effect of these factors on outcomes in injured children requiring intensive care. (Cassidy et al., 2013) Poor neighborhood and housing conditions may increase risk of severe traumatic injuries, including those resulting from accidental falls and violent injury. (Hutchings et al., 2016; Stolbach & Anam, 2017) There is a documented association between minority race and higher mortality rates in firearm injuries and pedestrian trauma (Rubenstein et al., 2019; Hamann et al., 2020). Public insurance status is associated with differences in pre-hospitalization triage patterns and post-hospitalization disposition. (Lee et al., 2017; Segui-Gomez et al., 2003) Taken together, these differences in injury risk factors and health care access may predispose disadvantaged children to higher injury severity, resulting in higher PICU utilization. Current literature supports the continued need to better understand the impact of social determinants of health on trauma-related outcomes to improve health equity among children.

The impact of social factors, including race, on a child’s likelihood of admission to the PICU setting following trauma is not well described. (Cassidy et al., 2013) To address this knowledge gap, the objective of this study was to describe the sociodemographic predictors of PICU admission following traumatic injury in a single center cohort study. Because of evidence of higher injury severity among US children of minority race, we hypothesized that these children would have higher rates of PICU admission and utilization.

## Methods

This was a retrospective study of children with a traumatic injury treated in a pediatric emergency department (ED) of a tertiary academic medical center which is also a Level 1 Pediatric Trauma Center. Children with significant burn injuries are generally cared for at the region’s burn center at a different medical center. Caring for over 400 injured children each year, our institution is the only Level 1 Pediatric Trauma Center in an urban region with a catchment area of approximately 470,000 children. (US Census Reporter, 2021). After obtaining approval from the University Hospitals Cleveland Medical Center institutional review board, children < 18 years of age treated at our center for an injury requiring activation of the pediatric trauma team’s response from July 1, 2011 through June 30, 2016 were identified using our center’s data from the Trauma Base (Clinical Data Management, Austin, TX) registry. We included children presenting to our pediatric ED as the initial site of care following a traumatic injury, as well as those transferred from other facilities—including clinics, urgent care centers, and other EDs—in the region for pediatric trauma care. In our center, the need for activation of either a “full” (Level 1) or “limited” (Level 2) pediatric trauma team response is determined by health care providers in the prehospital setting and/or the ED based on a set of established institutional criteria which include mechanism of injury, vital sign abnormalities, neurologic status, and required pre-hospital interventions (such as blood transfusion). Our institution’s pediatric trauma team is interdisciplinary and includes physicians and other health care providers with expertise in pediatric surgery, pediatric emergency care, pediatric critical care, pediatric radiology, and additional pediatric surgical subspecialties including neurosurgery and orthopedics. Children not requiring a full or limited activation of the pediatric trauma team response were excluded from the analysis.

For included children, we queried the Trauma Base for patient demographics, level of trauma team activation (“full” vs “limited”), general category of traumatic injury type (“blunt” vs “penetrating” vs “thermal”), presenting injury severity score (ISS), presenting Glasgow Coma Score (GCS), need for an operative procedure, and disposition (hospital admission, discharge to home, or death in ED prior to hospital admission). Additional data collected for children requiring hospitalization included length of hospital stay (LOS) in days, need for PICU admission at any time during the hospitalization, and rates of survival to hospital discharge.

The electronic health record was queried for data not available in our trauma registry including the mechanism of injury, reason for hospital admission, reason for PICU admission, presence of organ failure, PICU LOS in days, and type of operative procedure performed. Categories of injury mechanism included: pedestrian (for injuries involving a child struck by a motor vehicle); motor vehicle accident; fall (dichotomized to estimated height > 10 ft or < 10 ft); gunshot wound (including pellet gun injuries); recreational vehicle (including all-terrain vehicles, golf carts, motorized bikes and scooters, lawnmowers and tractors, and buggies); sports (including bicycle, snowboarding, skiing, sledding, skateboarding, football, wrestling and golf injuries); assault; other (including horse injuries, projectile injuries, stabbing injuries, crush injuries, burn injuries, dog bites, and unknown mechanism). Reason for hospital admission was categorized as “general care floor for observation,” indicating injury present not requiring a surgical intervention, but mechanism of injury warranted close vital sign monitoring, serial exams, and/or analgesic therapies to adequately control pain. Operative procedures were categorized as orthopedic, neurosurgical/spine, thoracic, abdominal, genitourinary/pelvic, vascular, facial/otolaryngologic, or wound care.

Our institution’s criteria for PICU admission specifies that children admitted to the PICU are at “risk of loss of vital functions [and] therefore, require a multidisciplinary special care unit designed to provide assessment, continuous observation, and interventions.” The reason for PICU admission was categorized as “at risk for organ failure,” for those children without evidence of organ failure, but whose mechanism of injury necessitated a higher level of nursing care (e.g. vital sign checks and exams every 1 to 2 h), or “organ failure,” for children who had evidence of end-organ failure. Examples of documented organ failure included encephalopathy defined by GCS < 15 or a trauma-associated seizure; a neurologic injury requiring a neurosurgical procedure and/or associated with a neurologic deficit; respiratory failure requiring oxygen support beyond that available on a general care floor; liver failure, renal failure, or pancreatic failure requiring surgical intervention; compartment syndrome requiring surgical intervention, or hemorrhagic shock requiring medical or surgical intervention.

Demographic data collected from the Trauma Base included patient age, gender, race, and residential ZIP code. For subjects with missing race data or classified as “other” race, race data from the electronic health record was used. For our analysis, race was categorized as White or racial minority, which included patients identified as Black, Hispanic ethnicity, Asian, Native American, or “other” race. Median household income for each patient was estimated by matching recorded patient ZIP codes to publicly available 2014 US Census Bureau data. In 2014, the designated federal poverty level for a family of four in the US was $23,850. (US Census Bureau, 2014) For this study, we designated children living in a ZIP code with a median household income less than the federal poverty level as “below poverty level,” and those children living above the federal poverty level as “above poverty level.”

The primary outcome measure for this study was rate of admission to a PICU setting for care following a traumatic injury. Other outcome measures included PICU LOS, hospital admission rate (regardless of need for PICU care), hospital LOS, and mortality. Analyses were conducted using SigmaPlot 12.5 (Systat Software, Inc., San Jose, CA). Descriptive analyses were used to interpret demographic data and are presented as proportions. Because the data were not normally distributed, Chi square or Mann Whitney rank sum tests were used, as appropriate, to compare differences in demographics, injury characteristics, patient disposition, mortality, hospital utilization and PICU utilization between minority and White children. Variables loosely associated (*p* < 0.1) with PICU admission in univariate analyses were included in the multivariate logistic regression analysis. For univariate analysis of PICU LOS, we dichotomized the variable based on duration longer than the 75% (> 2.5 days). (Basques et al., 2014) Variables associated (*p* < 0.1) with a prolonged PICU LOS were included in the multivariate linear regression model. Continuous data are presented as median values and interquartile ranges, while categorical data are presented as numbers and percentages.

## Results

During the 5-year study period, 2094 patient encounters were recorded in our institution’s trauma database. Six hundred fifty-four (31.2%) patients required a full or limited response by the pediatric trauma team and were therefore included in the analysis. Patient demographics and overall injury characteristics are summarized in Table 1. The median age of included children was 8.0 years [IQR 4.0–13.0], 434 (66.4%) were male, and 361 (55.2%) were categorized as minority race. More than 25% (*n* = 165) of children included in the analysis had an estimated household income based on ZIP code below the poverty level, with an estimated median household income of US $40,682 [24,065-53,339] for the entire cohort. Most children sustained an injury due to a blunt trauma mechanism (90.1%) as opposed to a penetrating or thermal mechanism. Overall, the injury severity in this cohort of children was low (median 4 [IQR 1–9]).

**Table 1:** Patient Demographics and Injury Characteristics by Race

	*Entire Cohort n = 654*	*Minority Children n = 361*	*White Children n = 293*	*p-value*
Age (*years*)^*^	8.0 [4.0–13.0]	8.4 [4.0–13.0]	8.0 [3.5–12.5]	p = 0.027
Gender (*male*)	434 (66.4%)	193 (65.9%)	241 (66.8%)	*p* = 0.876
Estimated Household Income Below Poverty Level (*yes*)	165 (25.3%)	152 (42.1%)	13 (4.4%)	*p* < 0.001
Trauma Activation (*full)*	102 (15.6%)	67 (18.6%)	35 (11.9%)	*p* = 0.027
Trauma Type				*p* < 0.001
Blunt	589 (90.1%)	308 (85.3%)	281 (95.9%)	
Penetrating	62 (9.5%)	50 (13.9%)	12 (4.1%)	
Thermal	3 (0.5%)	3 (0.8%)	0 (0.0%)	
Intentional Trauma (*yes*)	77 (11.8%)	64 (17.7%)	13 (4.4%)	*p* < 0.001
ISS^#^	4 [1–9]	2 [1–9]	5 [1–10]	*p* < 0.001
GCS^$^	15 [15–15]	15 [15–15]	15 [15–15]	*p* = 0.917
Mechanism of Injury^^^				*p* < 0.001
Pedestrian^**^	172 (26.3%)	133 (36.9%)	39 (13.3%)	
Motor Vehicle Accident	125 (19.1%)	65 (18.0%)	60 (20.5%)	
Fall (estimated < 10 ft)^**^	82 (12.5%)	27 (7.5%)	55 (18.8%)	
Gunshot Wound^**^	57 (8.7%)	50 (13.9%)	7 (2.4%)	
Fall (estimated > 10 ft)	54 (8.3%)	31 (8.6%)	23 (7.8%)	
Recreational Vehicle	50 (7.6%)	6 (1.7%)	44 (15.0%)	
Sports^**^	48 (7.3%)	14 (3.9%)	34 (11.6%)	
Assault	29 (4.4%)	19 (5.3%)	10 (3.4%)	
Other	31 (4.7%)	12 (3.3%)	19 (6.5%)	

*Abbreviations and Definitions: ISS* injury severity score, *GCS* Glasgow Coma Score, “Minority” defined as Black (*n* = 338), Hispanic ethnicity (*n* = 10), Native American (*n* = 1) or “other” (*n* = 12) race; “Below Poverty Level” defined as living in a ZIP code where the estimated median annual household income for a family of 4 is less than the 2014 federal poverty level (<$23,850); “Other” mechanism of injury included 9 subjects injured by a horse, 8 injured with a projectile (excluding bullets), 6 stabbing injuries, 5 crush injuries, 2 burn injuries, and 1 dog bite; ^*^2 subjects with missing age data; ^#^6 subjects with unknown ISS; ^$^6 subjects with unknown GCS; ^^^6 subjects with unknown injury mechanism; **mechanism of injury categories found to be different (*p* < 0.05) in post-hoc analyses; data presented as median [interquartile range] or *n* (%)

**Fig. 1:**
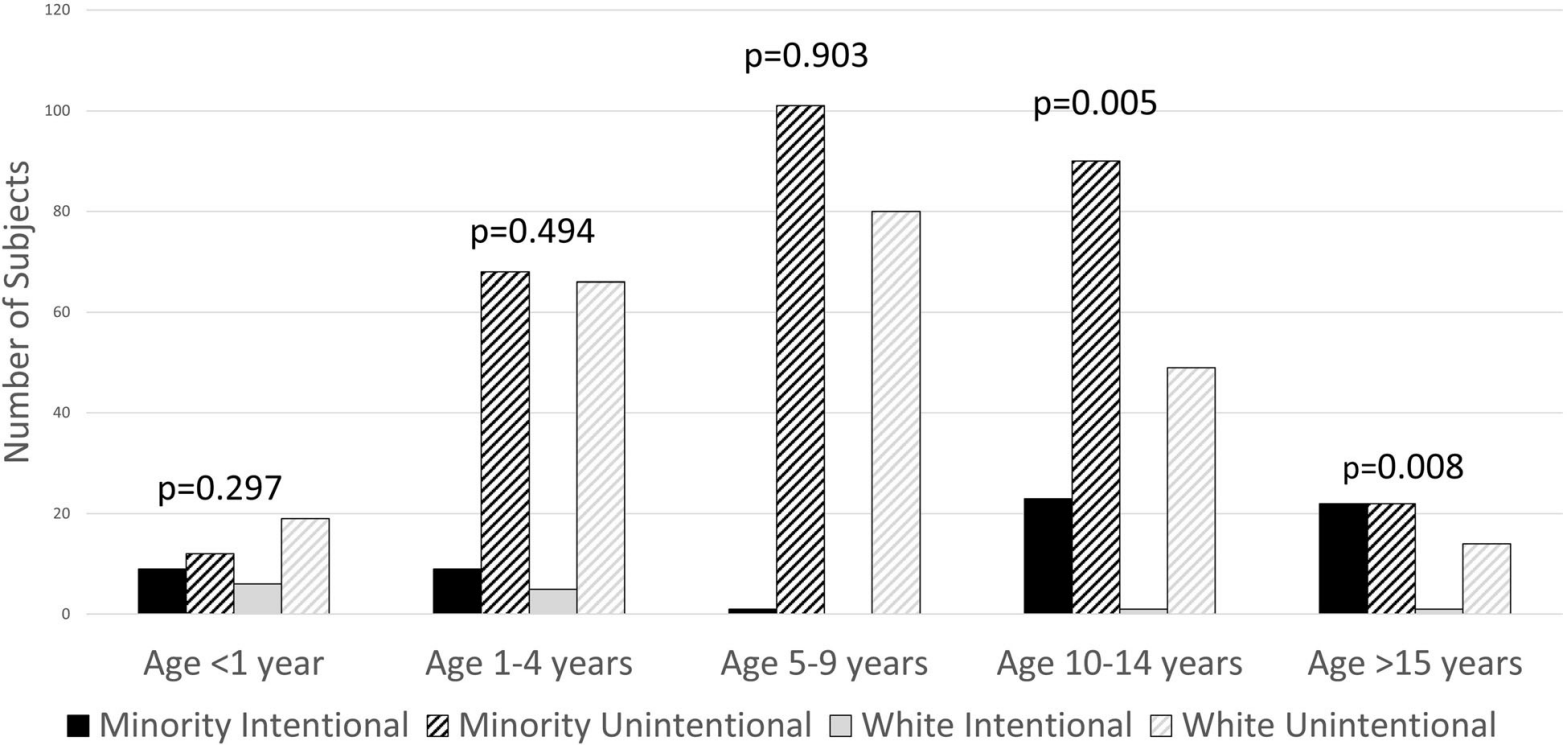
Differences in Injury Patterns Based on Age and Race

There were differences in injury patterns based on age and race categories (Table 1 and Fig. 1). Seventy-seven children suffered intentional injuries, including 42 (54.5%) gunshot wounds, 29 (37.7%) assaults, 5 (6.5%) stabbings, and 1 (1.3%) fall from a height > 10 ft. Overall, minority children were victims of intentional injury more often than White children (17.7% vs 4.4%, *p* < 0.001). The most common injuries in children < 1 year of age were falls < 10 ft (34.0%) and abuse/assault (29.8%). Pedestrian injuries were most common in children aged 1–4 years (20.8%), 5–9 years (35.3%) and 10–14 years (28.2%). Older teenagers (> 15 years) were most commonly injured from gunshot wounds (33.9%) There were also differences in injury mechanisms based on race. Minority children more frequently suffered pedestrian injuries (36.9% vs 13.3%, *p* < 0.001) and gunshot wounds (13.9% vs 2.4%, *p* < 0.001) compared to White children; White children were more likely to suffer injury from low height falls (18.8% vs 7.5%, *p* < 0.001) and sports injuries (11.6% vs 3.9%, *p* < 0.001) compared to minority children.

Children classified as having a mild injury (ISS < 9, *n* = 447) were more likely to be minority (73.4% vs 62.1%, *p* < 0.001) and children moderately injured (ISS 9–15, *n* = 144) were more likely to be White (30.0 vs 15.5%, *p* < 0.001). There was no difference in race among the severely injured children (ISS > 15, *n* = 57). Injured children who required full trauma team activation were more likely to be a minority race compared to children with limited trauma activation (65.7% vs 53.3%, *p* = 0.027). There were no differences in age, gender or percentage of children living in poverty between children classified as full or limited trauma.

Within this cohort of children, 9 (1.4%) died in the ED and 577 (88.2%) were admitted to the hospital (Table 2). The overall mortality was 2.8%. The median age of children who died was 3.8 [2.0–6.7] years, and the median ISS was 25.5 [19.8–75.0]. Most children who died were victims of assault (*n* = 4) and gunshot wounds (*n* = 4). Of the children requiring hospitalization, one-third (*n* = 195) were admitted to the PICU setting; 164 were admitted to the PICU directly from the ED, 26 were admitted following a surgical intervention in the OR, and 4 were admitted from the general care floor. Nearly one-quarter of hospitalized children required an operative procedure during their hospitalization. Categories of operative procedures included orthopedic (*n* = 49), neurosurgical/spine (*n* = 30), facial/otolaryngologic (*n* = 21), abdominal (*n* = 19), wound care (*n* = 10), thoracic (*n* = 7), vascular (*n* = 4), and genitourinary/pelvic (*n* = 1).

**Table 2:** Patient Disposition, Mortality, Hospital Utilization and PICU Utilization by Race

Patient Disposition^*^ and Mortality				
	*Entire Cohort n = 654*	*Minority Children n = 361*	*White Children n = 293*	*p-value*
Hospital Admission	576 (88.1%)	303 (83.9%)	273 (93.2%)	p < 0.001
Discharged to Home from ED	67 (10.2%)	49 (13.6%)	18 (6.1%)	*p* = 0.007
Died in the ED	9 (1.4%)	8 (2.2%)	1 (0.3%)	*p* = 0.087
Died in the Hospital	9 (1.4%)	6 (1.7%)	3 (1.0%)	*p* = 0.719
Overall Mortality	18 (2.8%)	14 (3.9%)	4 (1.4%)	*p* = 0.087
**Hospital Utilization**				
	*Hospital Cohort n = 576*	*Minority Children n = 303*	*White Children n = 273*	
Hospital LOS *(days)*^*#*^	1.0 [1.0–3.0]	1.0 [1.0–2.3]	2.0 [1.0–3.0]	*p* = 0.002
General Care Floor Admission for Observation (*yes*)	315 (54.7%)	190 (62.7%)	125 (45.8%)	
Operative Procedure^$^	128 (22.2%)	68 (22.4%)	60 (22.0%)	*p* = 0.973
Admitted to PICU setting	195 (33.9%)	79 (26.1%)	116 (42.5%)	*p* < 0.001
**PICU Utilization and Injury Characteristics**				
	*PICU Cohort n = 195*	*Minority Children n = 79*	*White Children n = 116*	
PICU LOS (*days*)	1.0 [0.7–2.5]	1.4 [0.7–3.9]	0.9 [0.7–1.9]	*p* = 0.032
Hospital LOS (*days*)^**^	3.0 [2.0–7.0]	4.0 [2.0–10.0]	3.0 [2.0–5.0]	*p* = 0.053
ISS in PICU Patients	9 [5.0–16.0]	10.0 [5.0–19.0]	9.0 [5.0–14.0]	*p* = 0.045
Mild Injury (ISS < 8)	72 (36.9%)	24 (30.4%)	48 (41.4%)	*p* = 0.158
Moderate Injury (ISS 8–10)	79 (40.5%)	29 (36.7%)	50 (43.1%)	*p* = 0.457
Severe Injury (ISS > 16)	44 (22.6%)	26 (32.9%)	18 (15.5%)	*p* = 0.007
Reason for PICU Admission				*p* = 0.007
At Risk for Organ Failure	98 (50.3%)	30 (38.0%)	68 (58.7%)	
Organ Failure	97 (50.0%)	49 (62.0%)	48 (41.4%)	

*Abbreviations and Definitions: PICU* pediatric intensive care unit, *ED* emergency department, *LOS* length of stay, *ISS* injury severity score; “Minority” defined as Black, Hispanic ethnicity, Native American, or “other” race; “operative procedure” included subjects requiring a surgical procedure in the operating room at any point during the hospitalization; “organ failure” defined as encephalopathy evidenced by GCS < 15 or a trauma-associated seizure; a neurologic injury requiring a neurosurgical procedure; respiratory failure requiring oxygen support unable to be provided on a general care floor; liver failure, renal failure, or pancreatic failure requiring surgical intervention; compartment syndrome requiring surgical intervention, or hemorrhagic shock requiring medical or surgical intervention; ^*^2 subjects transferred to different institution from the ED for care; ^#^2 subjects with missing LOS data; ^$^12 patients with multiple procedures; ^**^hospital LOS for patients requiring PICU care; data are presented as median [interquartile range] or *n* (%)

Differences in patient hospital utilization based on race were observed. Compared to White children, minority children were less likely to be admitted to the hospital, had shorter hospital LOS, and were less likely to be admitted to the PICU. However, among those children admitted to the PICU, minority children had higher ISS, higher rates of organ failure, and longer PICU LOS, compared to White children.

Demographics and injury characteristics associated with a PICU admission are shown in Table 3. After adjusting for age, level of trauma activation, trauma type, injury severity score, and need for a surgical procedure, minority children were still less likely to be admitted to the PICU compared to White children. The effect of neighborhood income, as estimated by patient ZIP code, was lost in the multivariable model (Table 4).

**Table 3:** Demographics and Injury Characteristics by PICU Admission

	*PICU Yes (n = 195)*	*PICU No (n = 381)*	*p-value*
Age (years)^*^	6.0 [2.1–12.0]	9.0 [5.0–13.3]	p < 0.001
Gender *(male)*	127 (65.1%)	260 (68.2%)	*p* = 0.596
Minority *(yes)*	79 (40.5%)	224 (58.8%)	p < 0.001
Estimated Household Income Below Poverty Level *(yes)*	29 (14.9%)	107 (28.1%)	p < 0.001
Trauma Activation *(full)*	64 (32.8%)	28 (7.3%)	p < 0.001
Trauma Type *(penetrating)*	19 (9.7%)	33 (8.7%)	*p* = 0.333
Intentional Trauma (*yes*)^#^	36 (18.5%)	41 (10.8%)	*p* = 0.013
ISS^$^	9 [5–16]	2 [1–5]	p < 0.001
Operative Procedure^^^	67 (34.4%)	61 (16.0%)	p < 0.001

*Abbreviations and Definitions: PICU* pediatric intensive care unit, *ISS* injury severity score; “Minority” defined as Black, Hispanic ethnicity, Native American, or “other” race; “Below Poverty Level” defined as living in a ZIP code where the estimated median annual household income for a family of 4 is less than the 2014 federal poverty level (<$23,850); ^*^2 subjects with missing age data; ^#^6 subjects with intentionality of trauma unknown; ^$^6 subjects with unknown ISS; ^^^2 subjects with unknown surgical data; data presented as median [interquartile range], *n* (%)

**Table 4:** Multivariate Model of Demographics and Injury Characteristics Associated with PICU Admission

*Demographics and Injury Characteristics*	*Coefficient*	*OR [95% C.I.]*	*p-value*
Age *(years)*	−0.122	0.885 [0.846–0.927]	*p* < 0.001
Minority (*yes*)	−0.709	0.492 [0.298–0.813]	*p* = 0.006
Estimated Household Income Below Poverty Level (*yes*)	−0.437	0.646 [0.343–1.218]	*p* = 0.177
Trauma Activation *(full)*	1.889	6.614 [3.143–13.918]	*p* < 0.001
ISS Score	0.188	1.207 [1.153–1.265]	*p* < 0.001
Intentional Trauma (*yes*)	0.003	0.997 [0.449–2.214]	*p* = 0.994
Operative Procedure	0.054	1.055 [0.614–1.816]	*p* = 0.845

*Abbreviations and Definitions: PICU* pediatric intensive care unit, *OR* odds ratio, *C.I.*, confidence interval; *ISS*, injury severity score; “Minority” defined as Black, Hispanic ethnicity, Native American, or “other” race; “Below Poverty Level” defined as living in a ZIP code where the estimated median annual household income for a family of 4 is less than the 2014 federal poverty level (<$23,850); variance inflation factor ranged 1.04–1.49 for independent variables

Demographics and injury characteristics associated with a prolonged PICU LOS are shown in Table 5. Minority children were more likely to have a prolonged PICU LOS in univariate analysis, but after adjusting for illness severity and characteristics, the association was lost (Table 6).

**Table 5:** Demographics and Injury Characteristics Associated with a Prolonged PICU Length of Stay

	*Prolonged PICU LOS Yes (n = 50)*	*Prolonged PICU LOS No (n = 145)*	*p-value*
Age (years)^*^	4.9 [1.0–12.3]	6.0 [2.3–12.0]	*p* = 0.358
Gender *(male)*	32 (64.0%)	95 (65.5%)	*p* = 0.982
Minority *(yes)*	31 (62.0%)	48 (33.1%)	*p* < 0.001
Estimated Household Income Below Poverty Level *(yes)*	9 (18.0%)	20 (13.8%)	*p* = 0.624
Trauma Activation *(full)*	30 (60.0%)	34 (23.5%)	*p* < 0.001
Trauma Type *(penetrating)*	11 (22.0%)	8 (5.5%)	*p* = 0.003
Intentional Trauma (*yes*)^#^	21 (42.0%)	15 (10.3%)	*p* < 0.001
ISS^$^	17.5 [9.8–22.8]	9 [4.0–11.0]	*p* < 0.001
Operative Procedure^^^	35 (70.0%)	32 (22.1%)	*p* < 0.001
Organ Failure (*yes*)	45 (80.0%)	52 (35.9%)	*p* < 0.001

*Abbreviations and Definitions: PICU* pediatric intensive care unit, *LOS* length of stay, *ISS* injury severity score, "Prolonged PICU LOS" defined as >2.5 days; “Minority” defined as Black, Hispanic ethnicity, Native American, or “other” race; “Below Poverty Level” defined as living in a ZIP code where the estimated median annual household income for a family of 4 is less than the 2014 federal poverty level (<$23,850); ^*^2 subjects with missing age data; ^#^6 subjects with intentionality of trauma unknown; ^$^6 subjects with unknown ISS; ^^^2 subjects with unknown surgical data; data presented as median [interquartile range], *n* (%)

**Table 6:** Multivariate Model of Prolonged PICU Length of Stay

*Demographics and Injury Characteristics*	*β-Coefficient*	*t-statistic*	*p-value*
Minority (*yes*)	−0.663	−0.987	*p* = 0.325
Trauma Activation *(full)*	1.720	1.979	*p* = 0.049
Trauma Type (*penetrating*)	−0.825	−0.706	*p* = 0.481
Intentional Trauma (*yes*)	2.850	3.087	*p* = 0.002
ISS	0.0302	0.960	*p* = 0.338
Operative Procedure	1.934	2.591	*p* = 0.010
Organ Failure (*yes*)	1.387	1.844	*p* = 0.067

*Abbreviations and Definitions: PICU*, pediatric intensive care unit; *OR*, odds ratio; *C.I*., confidence interval; *ISS*, injury severity score; “Prolonged PICU Length of Stay” defined as >2.5 days; “Minority” defined as Black or “other” race; r-squared for this model is 0.256

## Discussion

In this single-center study of children requiring trauma team activation at an urban tertiary pediatric center, racial minority was independently associated with lower odds of PICU admission, even after adjusting for other demographic factors and injury characteristics. However, for children admitted to the PICU—while minority children had higher injury severity— race was not an independent predictor of prolonged PICU LOS in this study. These findings were counter to our hypothesis that minority race would be associated with increased hospital and intensive care utilization following traumatic injury. Large multicenter database studies have demonstrated higher hospitalization rates and worse clinical outcomes, including mortality, among economically disadvantaged and marginalized children with traumatic injury. (Hakmeh et al., 2010; Pressley et al., 2007; Hayes & Groner, 2005)

Our study findings may be related to our hospital’s geographic location and general patient demographics, and its status as the only Level 1 Pediatric Trauma Center in the region. It should be noted that injured children older than 16 years of age requiring trauma team activation are generally cared for within our institution’s adult trauma center, which helps to explain the relatively low percentage (< 1%) of children in this age category in our cohort. Our center serves a US city with a high poverty rate and a high proportion of residents from racial minority backgrounds. (United States Census, 2020) Our regional demographic patterns match what is seen in many other American cities – with higher proportions of racial minority and poor families centered in urban neighborhoods (including those surrounding our hospital campus), and outlying suburban neighborhoods comprised of more White and higher-income families. The close proximity of our institution’s emergency department to neighborhoods with relatively large populations of racial minority and urban poor children, combined with a network of community hospitals equipped to care for children living in suburban neighborhoods with low to moderate injury severity, likely skewed the results of this single center study. The differences in injury mechanism based on race in this study are also emblematic of these demographic differences in the built environment, with minority children at higher risk for pedestrian injuries. Our results align with previous database studies showing higher rates of pedestrian injuries in minority populations. (Hamann et al., 2020)

Regionalized trauma systems were developed to ensure appropriate triage and transfer of the most seriously injured children. While transfer and admission decisions should be based on patient severity level and a need for subspecialized care, previous studies of children and adults have suggested that social factors may also impact these decisions. In a large, multicenter database study of over 4500 severely injured adults, after adjusting for multiple variables including demographics, injury mechanism, and injury severity, the absolute risk of admission to a non-trauma hospital facility versus transfer to a trauma center was 14.3% (95% CI, 9.2–19.4%) higher for patients with public insurance versus 11.2% (95% CI, 6.9–15.4%) higher for patients with private insurance, compared to patients without insurance. (Delgado et al., 2014) In a similarly designed pediatric study including more than 9000 traumatically injured children, those with public insurance had a higher odds of transfer to a dedicated trauma center from a referral hospital when compared to children with private insurance (odds ratio [OR]: 1.3; 95% CI: 1.0–1.5). (Huang et al., 2017) These studies suggest that non-clinical factors, including attempts to minimize revenue losses from treating underinsured patients, may influence triage decisions. (Delgado et al., 2014; Huang et al., 2017)

Our study results also suggest that non-clinical factors may have an impact on hospitalization rates and PICU utilization among injured children. Overall recorded injury severity scores and mortality for hospitalized children was low in this cohort, with the median ISS at 4, and median hospital LOS of 1 day. An ISS between 1 to 8 is considered “minor”, a score of 9–15 is “moderate,” and higher scores are classified as “severe”. (Baker et al., 1974) These data point towards a potential over-triage of children who may not require PICU care, or hospitalization at all. (Osen et al., 2010; Ciesla et al., 2008) Because various injury mechanisms are included in our set of activation criteria of both limited and full trauma team responses, we certainly captured children in this study who required trauma team activations due to a severe mechanism of injury, but were ultimately found not to have multiple and/or severe injuries. Another potential limitation of our study is that the ISS recorded in our trauma database may not accurately reflect the true severity of injury for all children included, as ISS is determined by database registrars based on documentation in the patient medical record (which, in some cases, may not have accurately reflected the true ISS).

During the study period, the death rate due to firearm-related injury in our state was 33.7/100,000 for Black teenagers, versus 1.9/100,000 in White teenagers. (Centers for Disease Control and Prevention, 2020) Although we found racial minority children in this study had lower ISS and lower rates of hospital admission, these children met criteria for full trauma team activation more frequently; they also had higher rates of penetrating trauma, including firearm-related, cutting, and piercing injuries. The patterns in our community of higher trauma-related deaths and penetrating trauma rates in racial minority children, and higher PICU utilization in White children reflects continued disparities in pediatric trauma-related health outcomes that certainly warrants further investigation. These differences in outcomes are undoubtedly multifactorial, related to neighborhood-level factors, systemic inequities in the healthcare delivery system including implicit biases, indiscriminate application of clinical pathways, and deficiencies in healthcare access. (Institute of Medicine (US) Committee on Understanding and Eliminating Racial and Ethnic Disparities in Health Care et al., 2003; Haider et al., 2013a; Haider et al., 2013b)

This study provokes many important questions, although some limitations of our study design preclude full elucidation of the exact answers. Does systematic racism impact decisions of patient disposition from the ED? Do socioeconomic factors influence the rates of hospitalization—including admission to a PICU setting—in severely injured children? Are current pediatric trauma care protocols adequate to ensure equitable health care delivery regardless of patient sociodemographics? Among the limitations to our study, the single-center design and small sample size limits the generalizability of our results. However, many US tertiary care children’s hospitals are located in urban neighborhoods with disparities and demographics similar to ours. Single-center studies such as this one may serve as a reference and encouragement for other centers to examine their referral patterns and hospital utilization based on sociodemographics. Area-based indicators of socioeconomic status are convenient, but the use of neighborhood ZIP codes and census data as a means of estimating patient household income could result in misclassification of patients’ socioeconomic status. Although previous investigations of ZIP code as a marker of socioeconomic status in large database studies have demonstrated adequate correlation with neighborhood-based deprivation, prospective studies that collect patient-reported socioeconomic data are needed before conclusions between estimated income and trauma outcomes are made. (Krieger et al., 1997; Braveman et al., 2005; Berkowitz et al., 2015) Insurance status was not captured and likely contributes to discrepancies in health care utilization patterns illustrated by this study. Dichotomization of children into 2 race/ethnicity categories over-simplifies the complex nature of healthcare inequities and discrimination. During our study timeframe, variability in how injury severity scores were documented in our center’s pediatric database was noted. Furthermore, ISS is likely an inadequate pediatric severity score, although it did independently predict need for PICU admission in this study. Missing data may affect interpretation of our results, and the retrospective study design precludes controlling for additional factors known to be associated with unfavorable hospital outcomes including patient presenting vital signs and co-morbidities. Future prospective studies could include collection of sociodemographic and environmental risk factors in traumatically injured children presenting to the ED.

## Conclusion

This retrospective study of traumatically injured patients found that racial minority children had a lower ISS and were less likely to require PICU admission than White children. While it is necessary to validate our findings in additional prospective studies, we believe that this study contributes to the current body of literature reflecting that disparities based on race persist in pediatric trauma-related care —including admission to the PICU setting and hospitalization overall—among children requiring activation of specialized pediatric trauma teams.

## Supplementary Information

**Additional file 1:.** Rainbow Babies & Children’s Hospital Pediatric Trauma Database worksheet.

## Data Availability

The trauma registry dataset analyzed during the current study are not publicly available because they contain protected health information including hospital admission and discharge dates, but a de-identified dataset may be available from the corresponding author on reasonable request.

## Abbreviations

PICU: Pediatric intensive care unit
ED: Emergency department
US: United States
ISS: Injury severity score
LOS: Length of stay
IQR: Interquartile range
OR: Odds ratio
C.I.: Confidence interval
