# Supplementary material for: US children of minority race are less likely to be admitted to the pediatric intensive care unit after traumatic injury, a retrospective analysis of a single pediatric trauma center
Source: Inj Epidemiol. 2021 Apr 12;8:14. doi: 10.1186/s40621-021-00309-x (PMC8040210; doi:10.1186/s40621-021-00309-x)
Supplement: Supplementary file 1 — Additional file 1:. Rainbow Babies & Children’s Hospital Pediatric Trauma Database worksheet. [file 40621_2021_309_MOESM1_ESM.pdf]

D/T DC

RAINBOW BABIES AND CHILDRENS HOSPITAL
PEDIATRIC TRAUMA ABSTRACT WORKSHEET

Trauma Number:______________________ Abstract Completed:____________________

Med.Rec #:____________________ NAME:____________________

CITY/ZIP:		RACE: W H I B A O	SEX: M F
INJURY TIME:	INJURY DATE		
HOSP. ARRIVAL TIME	HOSP. ARRIVAL DATE	DOB	AGE: D W M Y

CAUSE CODE: ____

DETAILS:____

____

____

____

____

____

____

____

LOCATION CODE:____

AGENCY NAME	SCENE SQUAD:	REFERRING SQUAD:
Notification Time		
Arrival at Scene Time		
Scene Departure Time		
Destination Arrival Time		
Destination	RBC 3010/3012 Ref. Hosp.	RBC 3010

Admit Source: 1 – Scene 3 – Out of State Hosp 2 – OH Hospital				Referring Facility:			
**1008 Ashtabula**	**1022 Elyria**	**1026 Geneva**	**1038 Tri-Point (East – Painesville)s**	**1005 Mercy Allen**		**1073 SWGH**	**1002 S.Pointe**
**1490 Ahuja**	**1023 Fairview**	**1028 Geauga**	**1042 Lake W - Will**	**1050 Metro**		**1077 SJWS**	**1095 Akron Kids**
**1012 Bedford**	**1094 Firelands**	**1051 Hillcrst**	**1044 Marymount**	**1061 Parma**		**1084 Trumbll**	
**1016 Conneaut**	**1025 Fisher-Titus**	**1039Lakewood**	**1098 Mercy Reg Lor**	**1065 Richmond**			**1999 UrgiCre**

**PROTECTIVE DEVICES** 0- Not used 1 – Used 2 – NA 3 - ND	5002 CHILD SEAT	5012 AIRBAG/BELT	5003 FLOTATION
	5006 HELMET	5010 SEATBELT	5011 UNKNOWN
	5013 AIRBAG	5014 YES, NOS	5007 NA
	5008 None	5001 Other, football gear, glasses, etc	

PEDIATRIC TRAUMA ABSTRACT WORKSHEET (page 2)

UNIT	ADMIT TIME	ADMIT DATE	DC TIME	DC DATE	DC TO UNIT
3010					
ED D/C Order ______					
IP D/C Order ______					3015

Team Notify Time		Level 1	Level 2 UP DWN

TEAM MEMBERS		ARRIVAL TIME	
Ped. Surg.Attending - PSA Boulanger Barksdale Kim Interim PSA Dingeldein			Anesthesia 90003 Y N
Chief Resident TCR	Name		Anes Arrival Time:
Peds ED Attending OR Fellow	Name		

VITAL SIGNS	SCENE 1	REFER 1.2	Ref. Transport 1.3	ED 2
RESP RATE				
PULSE				
SBP/DBP				
GCS EYE				
GCS VERBAL				
GCS MOTOR				
GCS TOTAL	15	15	15	15
Temperature				
Weight	P / K	P / K	P / K	P / K
O2 saturation				
Paralyzed	1- Intubate 2 Intub/parlay 3– Sedate 4-GCS legitimate 5- NA/not EMS			

Admitting Physician ADM

ConsSVC	Cons time	Cons Date
NSR		
ORT		
OMFS		
OPTHO		
ORAL/DENT		
PLAS		
PSA		
PSY		
UROL		

Cons SVC	Cons Time	Cons Date
CARD		
CPT		
ENT/OTO		
GI		
GYN/OB		
INFDIS		
NEU		

Vital Signs Hrly	Yes	No	Neuro Checks (GCS) Hrly	Yes	No

ICD-10 CODES:

____

____

____

**Abuse/Neglect** **Yes** **No**	**SBIRT: Y N N/A**
**IMMOBILIZED** SCENE(3032) ED(3010)	
**RADIOLOGY PROCEDURES/DATE/TIME**	**OTHER: PROVIDER DATE TIME(if in OR)**<br><br>50021 Gilmore 31 Son 01 Thomp 03 Hardesty 02 Liu<br>22 Getty 20002 Selman 20004 Bam 99994 NSR
CHEST TUBE / CPR	Defibrillation
TRACH CRIC	
VENT	

**ETOH**	NOT tested NEG	Tox results: 0- No 1- Yes 2 – NT 3- ND
**TOX:**	NOT tested - 2 NEG - 0 THC Coc Opi PCP Amp Barb Ben Oth	

Pre-Existing Conditions:

A01 Cardiac surg<br>A02 CAD	C01 Peptic ulcer, Pancreatitis, Infl bowel, cirrhosis, bilirubin >2% on admission	E00 Psych Disorder, ADD, ADDH, Depression, Bipolar	F01 HIV/AIDS<br>F02 Routine Steriods	J05 Chrnic Demyelint Dis	J09 CVA Hemiparesis	N01 Chr. Drug Abuse
A03 CHF<br>A05 MI			F03 Transplants<br>F04 Active Chemo	L04 Chr. Pulm. Cond., Asthma	J10 - Mental Retardation	N02 Chr. ETOH abuse
B01 DM-insulin depnd	D02 Coumadin therapy	D03 Hemophilia	D05 DVT/embolus	J06 Chr. Dementia	J04 Seizures	K00 - Obesity
B02 DM-nondepend II	D04 Pre-existing Anemia	D06 Sickle cell	I01 Rheum. Arthritis, Sys Lupus	J08 Parkinsons	M02 Dialysis (exc. Transplant)	P00 Pregnancy

**Autospy Performed**	Yes	No	Unk	NA	**Organ/Tissue Granted**	Yes	No	Unk	NA
**Organ/Tissue Req.**	Yes	No	Unk	NA	**Organ/Tissue Taken**	Yes	No	Unk	NA

PAYOR:______________________

COMPLICATIONS/COMMENTS:
